# A Novel Homozygous Truncating Mutation in *LAMB2* Gene in a Chinese Uyghur Patient With Severe Phenotype Pierson Syndrome

**DOI:** 10.3389/fmed.2019.00012

**Published:** 2019-02-04

**Authors:** Hong Tao Zhu, Mireguli Maimaiti, Chen Cao, Yan Fei Luo, Delihuma Julaiti, Lin Liang, Aizezi Abudureheman

**Affiliations:** Pediatric, First Affiliated Hospital of Xinjiang Medical University, Urumqi, China

**Keywords:** pierson syndrome, severe type, *LAMB2* gene, microcoria, homozygous mutation

## Abstract

**Objective:** Pierson syndrome (OMIM 609049) is a rare autosomal recessive disorder characterized by congenital nephrotic syndrome and complex ocular abnormalities. Severe renal symptoms had be associated with truncating mutations. Few Chinese patients from diverse ethnic background had been evaluated and reported with this syndrome. Here we report the first Uyghur patient with typical Pierson syndrome phenotypes and a novel pathogenic homozygous variant in *LAMB2* gene.

**Method:** A thirty-nine-day old Uyghur girl was born to consanguineous parents, the girl presented with general edema, severe hypotonia and bilateral microcoria. Laboratory tests revealed severe proteinuria, microscopic haematuria, hypoalbuminaemia. By the age of 74 days, she died of renal failure and respiratory infection. We detected on mutations of *LAMB2* gene by the sanger sequencing.

**Result:**Sanger sequencing detected a homozygous 2-bp deletion (c.2044_2045insTT/p.Cys682Phefs^*^13) in the exon 16 of *LAMB2* gene. Both parents are heterozygous carriers.

**Conclusion:** We reported the first Uyghur case of *LAMB2* gene homozygous mutation leading to severe phenotype Pierson syndrome. The clinical presentation of the patient and the novel pathogenic variant detected in this patient added to the overall knowledge of this rare condition.

## Introduction

Pierson syndrome (OMIM 609049) is a rare autosomal recessive disorder characterized by congenital nephrotic syndrome and complex ocular abnormalities. The first 2 patients with congenital nephrotic syndrome and peculiar eye abnormalities were described by Pierson et al. in 1963, Since then, 79 patients from about 60 families around the world have been diagnosed with Pierson Syndrome. Eighty seven percentage of patients exhibited symptom within the first 3 months of life (1). The prognosis of Pierson Syndrome is usually poor, and patients progress to end-stage renal disease in a few weeks or months (2). About 11 pathogenic variants had been reported in HGMD (accessed on Nov. 4th, 2017). Most of the variants are null variants. Few Chinese patients had been evaluated and reported with this syndrome. The so far reported three cases of Chinese Pierson syndrome patients are all Han Chinese, including two cases with infantile nephrotic syndrome and microcoria. The other two cases presented with isolated nephrotic syndrome (1, 3–5).

Here we report the first Uyghur patient with typical Pierson syndrome phenotypes and a novel pathogenic homozygous variant in *LAMB2* gene.

## Case Presentation

**Figure 1:**
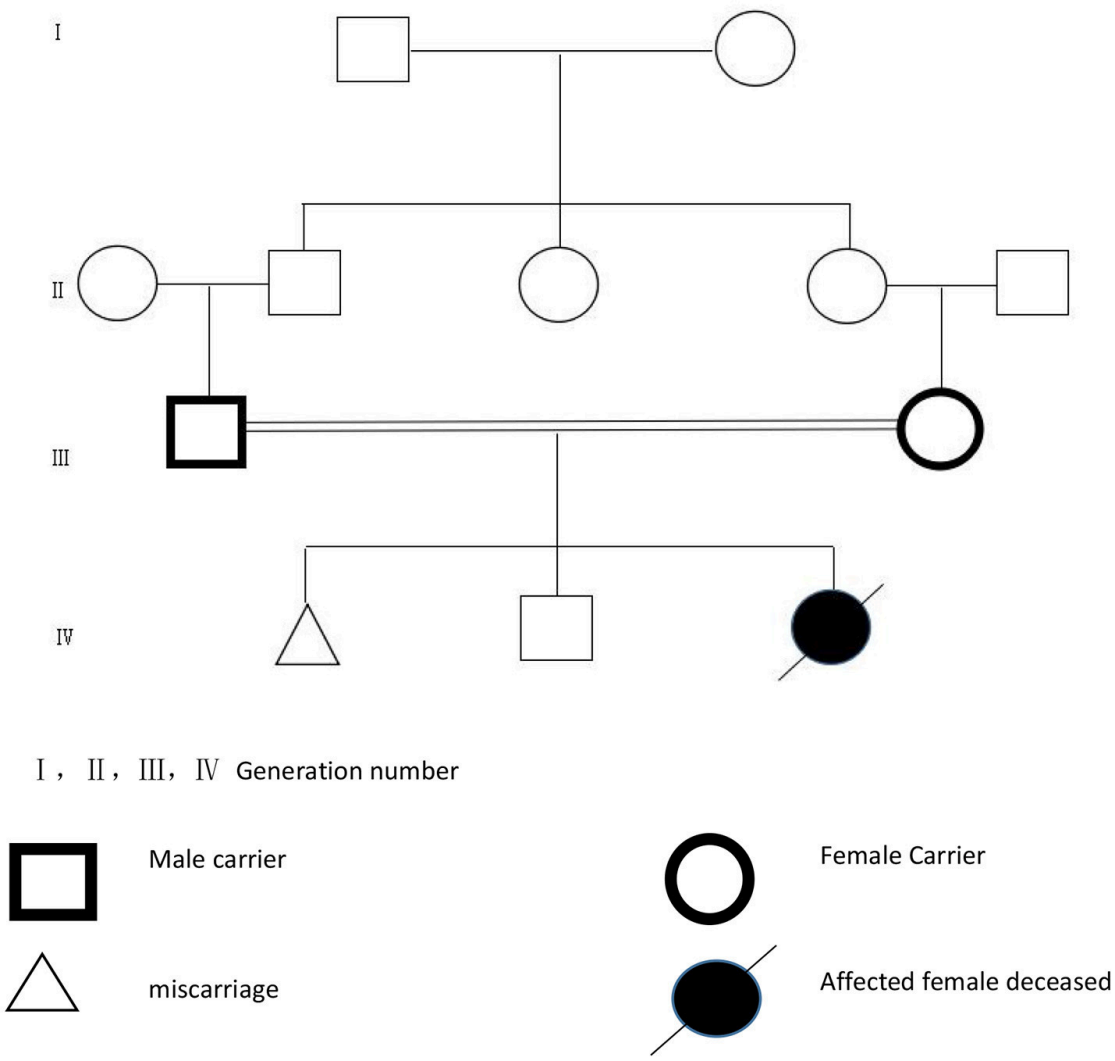
The pedigree.

A baby girl was born to consanguineous parents at 36 weeks of gestation by cesarean section. Her birth weight was 2,600 g. The mother, at 27 years of age, had two previous pregnancies, one ended as miscarriage at 16 weeks of gestation, the other led to a healthy boy now 5 (Figure 1). At the time of clinical evaluation at 39 days of age, the baby girl presented with general edema, particularly in the face, legs, and periorbital areas. She had severe hypotonia and ascites. The ocular exam revealed bilateral microcoria. Laboratory tests revealed severe proteinuria, microscopic haematuria, hypoalbuminaemia (5 g/L, normal range 34–48 g/L) and renal insufficiency(BUN9.73 mmol/L, normal range 2.9–8.2 mmol/L, Scr 200.05 μmol/L, normal range 53–115 μmol/L). Her electrolytes indicated severe hypokalemic and mild hypocalcemia. Serological tests were negative for syphilis, toxoplasmosis, rubella, cytomegalovirus (CMV), hepatitis B virus, hepatitis C virus, herpes virus, and HIV. Renal ultrasonography showed the size of the right kidney as 65 × 30 mm and the left kidney 60 × 29 mm. The patient was treated with intravenous rehydration, albumin infusion and peritoneal dialysis but her condition deteriorated gradually with serious edema and dyspnea. By the age of 74 days, she died of renal failure and respiratory infection.

## Materials and Methods

This study was conducted in accordance with the regulations of the national health and family planning commission of the People's Republic of China on the ethical review of biomedical research involving human beings to ensure the confidentiality and protection of data. After obtaining the written informed consent from the patient's guardian, the ethics committee of the first affiliated hospital of xinjiang medical university approved the agreement, and the subject's written informed consent was based on the “Declaration of Helsinki,” written informed consent was obtained from the participant for the publication of this case report.

Genomic DNA was extracted from patient's peripheral blood, Sanger sequencing for *LAMB2* was performed following the method previously described (1). Sixteen pairs of primers were designed covering all 32 exons and exon-intron boundaries of *LAMB2* gene using Primer 3.0 online. The PCR reaction was performed in a volume of 25 μl, comprising 12.5 μl 2 × Taq plus Master Mix (Tiangen Biotech Co., Ltd., Beijing, China), 1 μl 5 pmol/μl sense primer and antisense primer, and 1 μl 50 ng/μl DNA. The amplification was carried out under a touchdown PCR procedure with annealing temperature from 64° to 57°C, descending by 1°C every 2 cycles, 26 cycles until 57°C. PCR products were visualized with 2% agarose gel electrophoresis and sequenced bidirectionally with ABI 3730XL (SinoGenoMax Company Limited, China).

## Results

### Renal Pathology Findings

**Figure 2:**
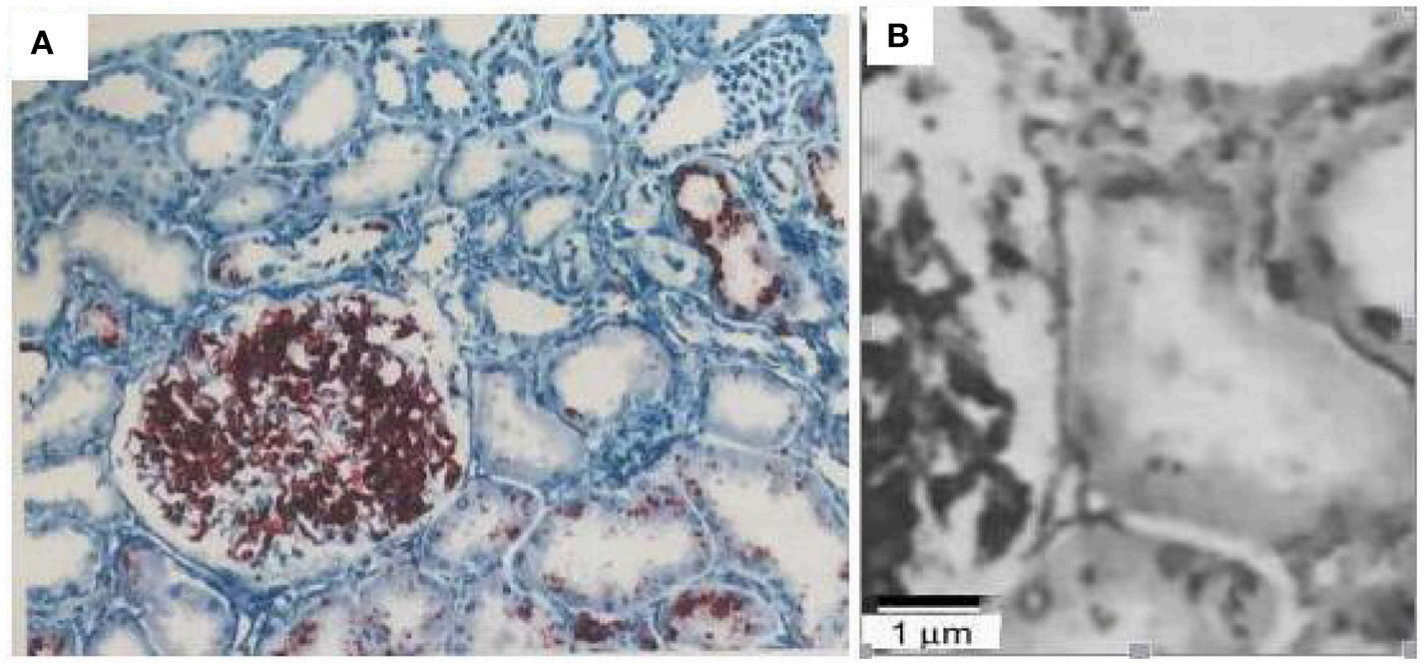
Renal biopsy findings of the patients with focal segmental glomerulosclerosis, mild tubular atrophy, and renal interstitial fibrosis. Scale bar is 40 um **(A)** and 1 um **(B)**.

The obtained renal biopsy specimen contained twenty-four significantly affected glomeruli, including four glomeruli with segmental sclerosis and six glomeruli with global sclerosis. Electron microscopy demonstrated focal adhesion of the GBM, and diffused swelling of podocyte. The pathological diagnosis was focal segmental glomerulosclerosis, mild tubular atrophy and renal interstitial fibrosis (Figure 2).

### Mutational Analysis

**Figure 3:**
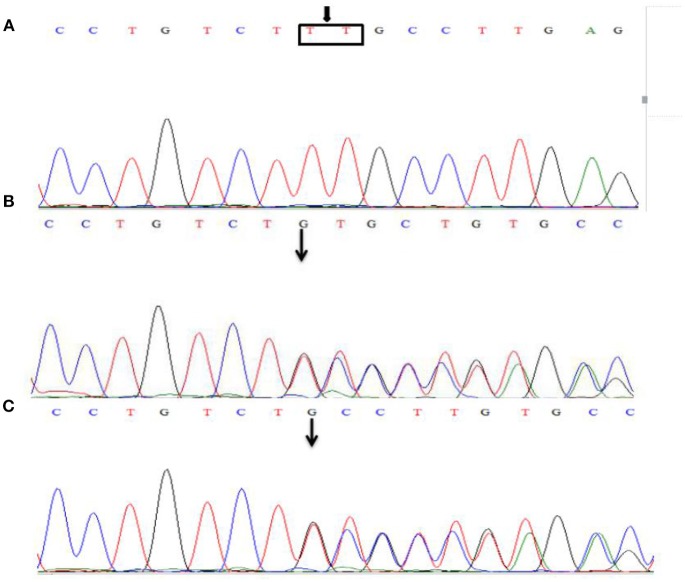
Sanger sequencing of the *LAMB2* gene. The patient was identified with 2-bp deletion; c.2044_2045insTT **(A)**, NM_002292.3, chr3:49163904-49163905. Both parents are heterozygous carriers **(B,C)**, and the arrows show the position of the mutation.

Sanger sequencing detected a homozygosis 2-bp deletion (c.2044_2045insTT/p.Cys682Phefs^*^13) in the exon 16 of *LAMB2* gene, the frameshift variant lead to a premature stop codon at 13 amino acid downstream. Both parents are heterozygous carriers (Figure 3). This variant has not been reported in the databases of HGMD and ClinVar.

## Discussion

*LAMB2* is the only gene currently known to be responsible for Pierson syndrome. Our patient exhibited typical clinical presentations of Pierson syndrome which include microcoria, general edema, and severe hypotonia. Single gene sequencing revealed a homozygous loss of function variant, which is consistent with null homozygous or compound heterozygous mutations previously report in patients with Pierson syndrome. The mutation detected in this patient is novel, it is located in the 16th of the 32 exons. It is predicted to lead to non-sense mediated decay. *LAMB2* encodes lamininβ2, a protein with 1,798 amino acids (6). Laminin β2 is a component of certain laminins, a family of heterotrimeric extracellular glycoproteins each consisting of α, β, and γ subunits joined together through a coiled coil. They participates in cell differentiation, adhesion, migration, and neuronal growth. Lamininβ2 is expressed in the glomerular basement membrane (GBM), ocular structures and neuromuscular synapses. The GBM which consists of three main components: capillary endothelium, GBM, and filtration fissures formed by the digital foot process of its interior (2), prevents blood plasma proteins from leaking into the urine, while allowing water, and small molecules to penetrate the barrier. Defect of or damage to any of these three layers can lead to proteinuria (7). Severe changes to GBM could lead to renal failure in the early stages (1). The patient we reported in this article developed a massive proteinuria, general edema, hypoalbuminemia, and hyperlipidemia after birth. We suspect that it is the disruption of the Laminin synthesis that affecting the function of the GBM.

Typical renal pathology of Pierson syndrome is the type of diffuse mesangial sclerosis, it can also present as focal segmental glomerulosclerosis or mesangial proliferative glomerular lesions. Electron microscopic examination revealed irregular contour of the GBM, thickening and layering of partial GBM and diffuse effacement of the foot processes (1). Other pathological changes may be related to the onset age and severity of the clinical symptoms. According to Kagan et al. for example, reported morphological findings in a patient with a milder variant of Pierson syndrome. A Light microscopic appearance of glomeruli showed no abnormalities (8). Rene et al. described a newborn infant who was presented with congenital nephrotic syndrome and renal insufficiency, as well as bilateral microcornia. Renal biopsy showed extensive diffused mesangial sclerosis (9). According to the histopathological findings of this report, our patient demonstrated focal segmental glomerulosclerosis.

The most characteristic ocular anomaly of the Pierson syndrome is microcoria. A wide range of additional abnormalities were found, including cataract, nystagmus, high myopia, and retinal abnormality (2, 6). Similarly, a laminin β2 chain deficiency results in aplasia or hypoplasia of the dilator muscles of the iris. The patient in our case had microcoria, the typical presentation of Pierson syndrome. Reference to previous studies shows that not all patients with Pierson syndrome have microcoria. There were three Pierson cases which did not report microcoria. The first case is a 55-day boy with 2,000 degrees of high myopia who had a homozygous missense mutationc. 499G > T, of *LAMB2* gene (8). The second is Hyun Jin Choi et al. who reported two cases of Pierson syndrome, one of which suffered retinal detachment in the right eye at 11 months of age and the gene mutation was c.536c > T, c.2283-2286 delCTCT (10). Qiu et al. reported a 34-day girl from China with c.1176-1178delTCT mutation and no microcoria (3). The typical Pierson syndrome is a severe multiorgan *LAMB2*-associated disorder, but there is a wide variation in the ocular phenotype. Some patients are diagnosed with Pierson syndrome from the early manifestation of the eye, while others had mild or no ocular symptoms, which only presented themselves at later stages. Therefore, renal function should be tested in patients presenting the ophthalmic features of *LAMB2*-associated disorders, in particular marked microcoria. Similarly, thorough ophthalmologic examination is recommended in any infant presenting nephrotic syndrome of unknown origin (11).

There are various genotypes and phenotype studies to date. In 2006, Hasselbacher et al. stated that patient with the frame-shift mutation of *LAMB2* gene (e.g., truncating mutations) can lead to the typical characteristic of Pierson syndrome, including neonatal renal failure, severe ocular abnormalities, and neurologic impairment in long-term survivors. However, patient with the missense mutation of *LAMB2* gene may display variable phenotypes ranging from a milder variant of Pierson syndrome to isolated congenital nephrotic syndrome (12). In fact, In 2016, Boutaina Zemrani et al. concluded that patients with mild phenotypes harbor at least one missense or non-truncating deletion mutation, The kidney pathology of this patient is usually shown minor glomerular changes or focal and segmental glomerulosclerosis, whereas patients with severe phenotypes have truncating mutations. They had severe renal symptoms or end-stage renal disease in the first year of life (10, 12–14).

Age of onset of kidney involvement, age of onset of end-stage renal disease, and extrarenal presentations are used to distinguish the mild and severe phentype of Pierson syndrome. We report a severe phenotype patient presented with typical Pierson syndrome developing to end-stage renal disease within the first year of life. The truncating mutation causes severe type of Pierson syndrome. Boutaina Zemrani et al. report a case of severe Pierson syndrome in an infant, which was a mutation of homozygous *LAMB2* gene truncated mutation, c. 2890 c > T. There were 964 amino acids from peptide synthesis of coding *LAMB2* gene. The baby had a severe clinical phenotype, and died of renal failure 7 days after birth (13). Another case report described by Kino et al. was an infant with truncating mutation exon 30 c.5077_5078insCCAG,exon 9 c.1225 +1 G > A, which presented with severe Pierson syndrome and progressed rapidly to renal failure death at 27 months (15). In recent years, with the improvement of gene detection technology, the number of cases of Pierson syndrome has increased. There were some undefined relations between phenotype and genotype. Missense mutations do not always exhibit mild phenotypes, and truncating mutations does not always produce severe phenotypes. The genotypes of these two cases were truncating mutations with mild clinical symptoms. The first case of Pierson syndrome in China was only presented with proteinuria at the age of 3, and renal functions were normal (1). In another case of 6-year-old patient from South Korea, renal functions were also normal (10). It has been reported that the same truncating mutations showed different phenotypes, there may be other factors affecting clinical phenotypes that need further study.

We reported the first Uyghur case of *LAMB2* gene homozygous truncating mutation that led to severe phenotype Pierson syndrome. The patient had early onset renal failure and treated with intravenous rehydration and albumin infusion. By the age of 74 days, she died of renal failure and respiratory infection. The homozygous truncating mutation detected in this patient is associated with severe phenotype. The clinical presentation of the patient and the novel pathogenic variant detected in this patient added to our knowledge of this rare condition.

## Author Contributions

HZ and CC are mainly responsible for the article writing. MM is the corresponding author. YL and DJ are responsible for clinical data analysis. LL and AA are responsible for collect patient data.

### Conflict of Interest Statement

The authors declare that the research was conducted in the absence of any commercial or financial relationships that could be construed as a potential conflict of interest.
